# Between a Rock and a Hard Place: Habitat Selection in Female-Calf Humpback Whale (*Megaptera novaeangliae*) Pairs on the Hawaiian Breeding Grounds

**DOI:** 10.1371/journal.pone.0038004

**Published:** 2012-05-29

**Authors:** Rachel Cartwright, Blake Gillespie, Kristen LaBonte, Terence Mangold, Amy Venema, Kevin Eden, Matthew Sullivan

**Affiliations:** 1 California State University Channel Islands, Camarillo, California, United States of America; 2 The Keiki Kohola Project, Lahaina, Maui, Hawaii, United States of America; 3 UCSB Library, University of California Santa Barbara, Santa Barbara, California, United States of America; 4 Manchester Metropolitan University, Manchester, United Kingdom; University of Western Ontario, Canada

## Abstract

The Au'au Channel between the islands of Maui and Lanai, Hawaii comprises critical breeding habitat for humpback whales (*Megaptera novaeangliae*) of the Central North Pacific stock. However, like many regions where marine mega-fauna gather, these waters are also the focus of a flourishing local eco-tourism and whale watching industry. Our aim was to establish current trends in habitat preference in female-calf humpback whale pairs within this region, focusing specifically on the busy, eastern portions of the channel. We used an equally-spaced zigzag transect survey design, compiled our results in a GIS model to identify spatial trends and calculated Neu's Indices to quantify levels of habitat use. Our study revealed that while mysticete female-calf pairs on breeding grounds typically favor shallow, inshore waters, female-calf pairs in the Au'au Channel avoided shallow waters (<20 m) and regions within 2 km of the shoreline. Preferred regions for female-calf pairs comprised water depths between 40–60 m, regions of rugged bottom topography and regions that lay between 4 and 6 km from a small boat harbor (Lahaina Harbor) that fell within the study area. In contrast to other humpback whale breeding grounds, there was only minimal evidence of typical patterns of stratification or segregation according to group composition. A review of habitat use by maternal females across Hawaiian waters indicates that maternal habitat choice varies between localities within the Hawaiian Islands, suggesting that maternal females alter their use of habitat according to locally varying pressures. This ability to respond to varying environments may be the key that allows wildlife species to persist in regions where human activity and critical habitat overlap.

## Introduction

Emerging research indicates that marine mammals fulfill a range of crucial functions within marine systems, from serving as bio-indicators of the health of marine eco-systems [1], [2] to the maintenance of key nutrient levels in surface waters in temperate marine regions [3], [4]. Consequently, the conservation of recovering marine mammal populations may be seen as more than just the protection of individual, charismatic species; pro-active management may be more broadly justified as a means of restoring and maintaining healthy marine ecosystems.

As many marine mammal populations typically aggregate to feed and to breed, protected area management can be highly effective [5]. However, where these aggregations coincide with areas of high anthropogenic activity and/or the aggregations themselves attract high levels of human activity in the form of eco- and wildlife tourism, effective management can be challenging. In regions where human activity and wildlife habitat overlap, alterations in animal behavior are commonly reported. In some cases the outcome may be beneficial; for example, in North American elk (*Cervus elaphus*), the use of urban areas reduces predation and provides alternative winter forage, resulting in increased reproductive success [6]. More typically though, a range of different mechanisms results in reduced individual fitness: in big horn sheep (*Ovis canadensis*), foraging in urban areas leads to increased parasite loads [7], in pronghorn antelope (*Antilocapra americana*), increased vigilance close by roadways reduces foraging time [8] and in forest elephants (*Loxodonta cyclotis*), individuals forego access to high quality food and habitat resources in response to human presence within favored areas [9]. The collective concern in all these studies is that in the absence of ameliorative management, changes in individual fitness may ultimately lead to population level impact.

In marine mammals that haul-out to breed, such as pinnipeds, fidelity to land-based breeding sites is typically high and human presence in these regions can lead to reductions in reproductive rates (e.g., [10]). However for entirely aquatic marine mammals, the fluid nature of their distribution means that deleterious changes in their environment initially lead to altered patterns of habitat use rather than changes in survival and reproductive success [11], [12]. Still, changes in distribution and habitat use are recognized as a consequence of disturbance and as potential pre-cursors to ecological changes and population level impacts [13]. Patterns of habitat use in mobile marine mammals therefore warrant close attention. In this study, we examine patterns of habitat use in female-calf humpback whale (*Megaptera novaeangliae*) pairs in the waters of the Au'au Channel, Hawaii, where high levels of human activity and critical humpback whale habitat overlap.

Wintertime congregations of humpback whales in Hawaiian waters comprise around 50% of the Central North Pacific Stock and number between 8,500–10,000 animals [14]. The Au'au Channel, between the islands of Maui, Lanai and Molokai, is the most populous region within Hawaii [15], [16] and is used preferentially by maternal females (i.e. lactating females with an accompanying calf) [17]. The channel also comprises a busy marine thoroughfare and serves as the center for a wide range of tourist-based ocean activities, including a flourishing whale watching industry. As the channel is a core region of the Hawaiian Islands Humpback Whale National Marine Sanctuary, some management restrictions are in place: federal guidelines mandate minimum approach distances of 90 m throughout Hawaiian waters and certain types of vessels (parasail and personal watercraft) are banned from most coastal waters during whale season each year. In the most recent reviews, the health status of humpback whales in the area is rated as fair and declining [18], with vessel interactions, ranging from strikes to impacts on behavior, identified as a primary cause of this decline. Notwithstanding, overall numbers are increasing and current estimates of annual population growth rates for this stock range between 6–8.1% [14], [19].

Wintertime migration of humpback whales from high latitude feeding areas to low latitude breeding grounds such as Hawaii is primarily seen as an anti-predator strategy for maternal females [20], [21]. Predatory pressure from killer whales (*Orcinus orca*) typically targets first season calves [22], but as low latitude breeding grounds lie beyond the killer whale's habitual range [23], the threat of predation is postponed until the calves' natal migration to the feeding grounds [22]. By this point, larger body size [24], and better co-ordination skills [21] reduce calf susceptibility to predation, however development of these attributes may be constrained by the energy budget of the female-calf pair. High energetic costs are associated with calving and lactation [25], and as maternal females fast throughout the breeding season, stored maternal fat comprises the sole source of nutrition for the female and her calf during this period [24]. Consequently, maternal behavioral strategies that conserve energy carry fitness benefits [26], as conserved energy resources become available to promote the growth and development of the maturing calf. Energetically conservative maternal strategies include a preference for protected coastal waters [27]–[34], where calm surface conditions may reduce energy consumption during swimming for young calves [33], [34]. Additionally, maternal females may use shallow and/or coastal waters to segregate from actively breeding adults [27], [28], [30], thereby minimizing the likelihood of energetically expensive associations with multiple male groups [27], [35].

In certain well-known odontocete populations, high levels of vessel activity or whale-watching have led to energetic stress and the subsequent abandonment of previously favored habitat [36], [37]. For mysticetes, while short term behavioral changes in response to vessel activity that would incur energetic costs have been documented (examples include increases in swimming speeds [38] and increased surface activity [39]), surprisingly few studies document patterns of habitat use within perturbed regions (see [40], [41] for exceptions). Arguably, the behavioral changes reported may not be biologically significant for most individuals in the population [42] and therefore they are unlikely to drive changes in habitat use. However for maternal females during the lactation period, the consequences of disruptions to the finely balanced energetic budget could include reduced calf body size [43] leading to increased susceptibility to predation during the natal migration [24]. Smaller adult body size may also lower adult fitness [44]. Additionally, poor quality natal habitat and increased stress during early development may carry lifelong physiological costs [45]–[48].

Currently, fine scale trends in maternal habitat choice within the Au'au Channel are poorly documented. The most frequently cited studies describing maternal habitat preference in the region were conducted in the late seventies, at which time female-calf pairs were reportedly seen in near shore waters in several localities across Hawaii [49] and within 0.5 km of the shoreline along the coast of West Maui [50]. Subsequent accounts of habitat use by female-calf pairs indicated some dispersal from the Maui shoreline between 1980 and 1984 [51], [52] and based on these accounts, the State of Hawaii implemented the current wintertime ban on thrill craft in near-shore waters. Follow up studies have not been conducted in the Au'au Channel since the ban was imposed in the early nineties, however quantitative studies in lesser-used female-calf habitat along the Kohala shoreline of the Big Island of Hawaii [53], [54] and the northern shoreline of Kauai [55], indicate that female-calf pairs favor shoreline waters in these areas and in the absence of updated information, these findings are applied ubiquitously across Hawaiian waters. Scientists and management agencies currently cite a consistent preference in female-calf pairs for shallow, coastal waters across Hawaii (e.g. [18], [56], [57]).

Accurate and up to date documentation of habitat preference is a prerequisite for effective management of critical habitat and this is especially important in coastal regions, where vessel traffic, human activity and wildlife may be concentrated. In this study, we use quantitative survey techniques to document current trends in habitat choice in maternal humpback whales using the more heavily trafficked eastern portions of the Au'au Channel. As humpback whale calves are followers [58] and typically remain within one body length of the maternal female throughout the breeding season [59], we document locations of female-calf pairs and attribute the choice of these locations to the maternal female. We compare levels of habitat use to habitat availability according to depth, distance from shore, underwater terrain and proximity to a local small boat harbor, Lahaina Harbor, and test the alternate hypothesis that maternal females preferentially use shallow, inshore waters. We demonstrate that maternal females avoid these waters and therefore we evaluate the roles that different environmental factors may play in shaping maternal habitat use in this area. Additionally, we examine current patterns of social stratification in this region, and based on these findings we speculate that current trends in maternal habitat use represent a trade-off between the potential benefits and varying pressures within this favored breeding region.

## Results

### Survey Data

**Figure 1:**
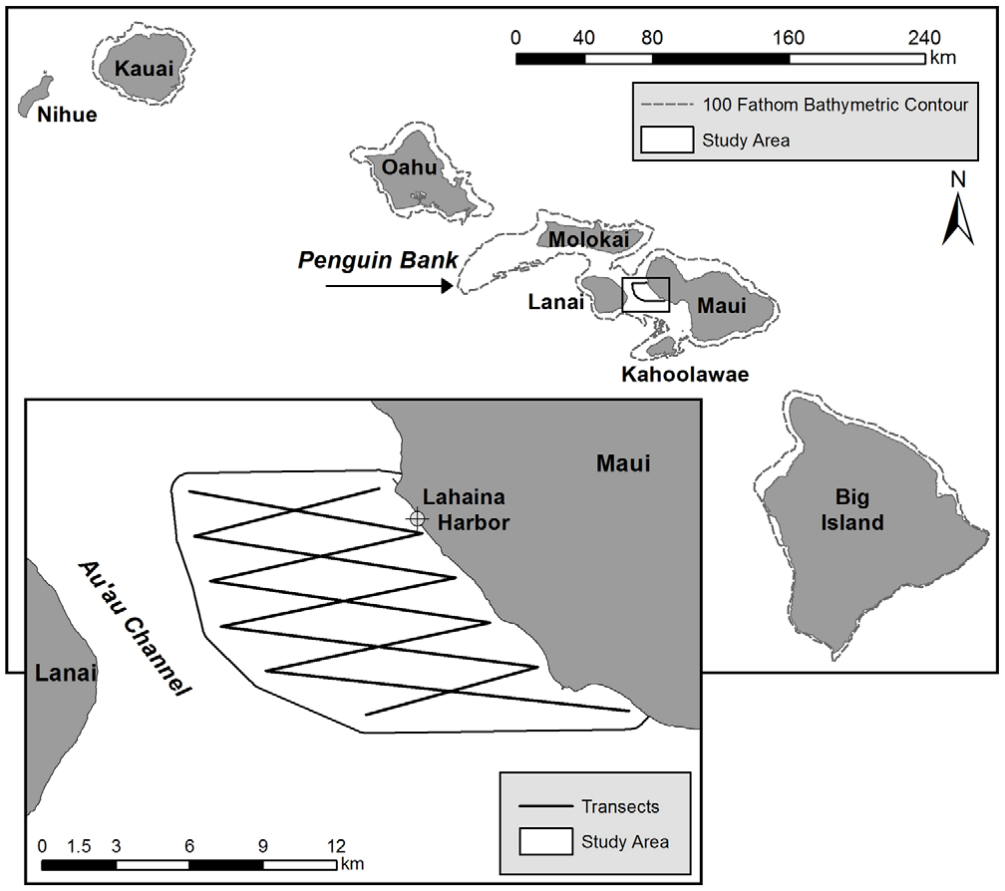
Map of the Hawaiian Islands, Au'au Channel and the study area. Within the study area, zigzag transect lines were constructed between waypoints set at 1 minute intervals. Inner waypoints were set at 0.25 km from the shoreline and outer way points set at the deepest or mid-point of the channel, whichever lay furthest offshore. The final perimeter of the study area extended from the shoreline to 750 m beyond the outer waypoints.

The chosen study area comprised the eastern portion of the Au'au Channel and included the shoreline of West Maui, Hawaii (Figure 1). It covered 124. 5 km^2^, extending from the shoreline to either the mid– or deepest point of the Au'au Channel at each minute of latitude, whichever lay furthest off-shore. Within the study area, water depths range up to 112 m, with a mean depth of 55.9 (s.d. 20.8) m. The terrain of the channel (the bottom topography) comprises ridges of drowned coral reef, referred to in this study as rugged regions, interspersed between flat, sandy, concentric basins, hereafter referred to as flat regions [60]. Transect–based surveys were conducted across the study area on 34 different days between 2008 and 2010, comprised of 177 hours of observation and covered a total distance of 731.1 km along randomly chosen survey lines that zigzagged across the study area. The locations of 148 groups that included 356 animals were included in the final dataset. The overall encounter rate for the entire study was 0.49 individual whales and 0.20 groups of whales km^−1^ (Table 1).

**Table 1:** Encounter rates for March ′08, ′09 and ′10, Au′au Channel, Maui, Hawaii.

Year	Number of individual whales sighted (n of groups)	Transects completed	Distance traveled (km)	Encounter rate (whales km^−1^) (groups km^−1^)
2008	113 (44)	27	270.5	0.417 (0.16)
2009	179 (77)	34	355.3	0.503 (0.22)
2010	64 (27)	10	105.3	0.607 (0.27)
Total Counts	356 (148)	70	731.1	0.487 (0.20)

As there was no evidence of inter-annual variability in sighting locations, data from the three consecutive years (2008–2010) were used in the study, (For distance from shore; ANOVA F_2,145_ = 2.397, *P* = 0.095, for water depth; F _2, 145_ = 0.385, *P* = 0.681, for proximity to the harbor; Kruskal – Wallis *X* ^2^ *_2, 148_* = 3.275, *P* = 0.194 and for nature of terrain; Pearson *X* ^2^ *_2, 148_* = 0.705, *P* = 0.703). A power analysis confirms the reliability of these results (For ANOVA; (1-β) = 0.775, for *X* ^2^ tests; (1-β) = 0.914, G-power [61]).

### Vessel distribution in the study area

**Figure 2:**
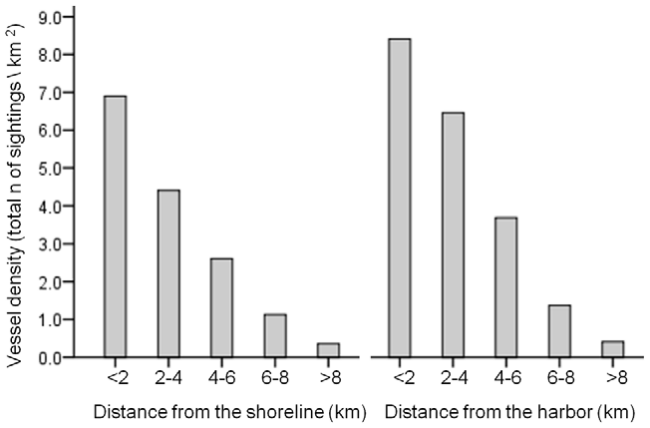
Shore-based estimates of vessel density in the eastern portion of the Au'au Channel. Differences between regions were significant for vessel density vs. distance to shoreline (*X* ^2^_4, 335_ = 174.1, p = <0.001) and for vessel density vs. proximity to the harbor (*X* ^2^_4, 335_ = 219.693, p<0.001).

A snap-shot survey of vessel traffic across the study area was conducted to identify key trends in vessel traffic levels within the study area. From a land-based survey site, 89.4 % of the study site (111.3 of 124.5 km^2^) was in view and only a small, inshore region to the south of the study site was out-of-view. During twice daily scans at varying times of the day, we documented the locations of 335 vessels in the study area, with a mean vessel sighting rate of 14.6 (s.d. 6.2) vessels per scan across the study site. Commercial, permitted vessels accounted for 84.5% of all vessels sighted. Vessel densities (sightings km^2^) peaked in shoreline regions and in regions closest to the harbor, and then declined with increasing distance from the shoreline and the harbor (Figure 2). The gradient of this decline was most pronounced in relation to harbor proximity and changes in vessel density were closely correlated with increasing distance from the harbor (r = −0.9). On the basis of these results we considered proximity to the harbor as a proxy for relative levels of vessel traffic within the study area.

Vessel sighting rates did not vary with time of day (for morning, mid-day and afternoon sighting rates; Kruskal Wallis *X^2^*_2, 335_ = 0.449, *P* = 0.978, 1–β = 0.998). When vessels were classed according to activity (whale-watching, transiting or stationary), the proportions of vessels engaged in different activities showed no evidence of variation either according to distance from shore (Pearson *X^2^_8, 335_* = 11.857, *P* = 0.158) or proximity to the harbor (Pearson *X^2^_8, 335_* = 9.371, *P* = 0.312).

### Trends in habitat use in female-calf pairs

A total of 86 female-calf groups, (i.e. groups containing a maternal female, her associated calf and any other number of adults) were encountered during the survey. The mean distance from shore for the locations of these groups was 4.75 (s.d. 2.27) km and the mean depth of water was 58.8 (s.d. 14.7) m. Mean proximity to the small boat harbor (Lahaina Harbor) was 5.94 (s.d. 1.99) km and 63% (54 of 86) groups were sighted in regions of rugged terrain (where bottom topography principally comprised of drowned reef [60]).

**Figure 3:**
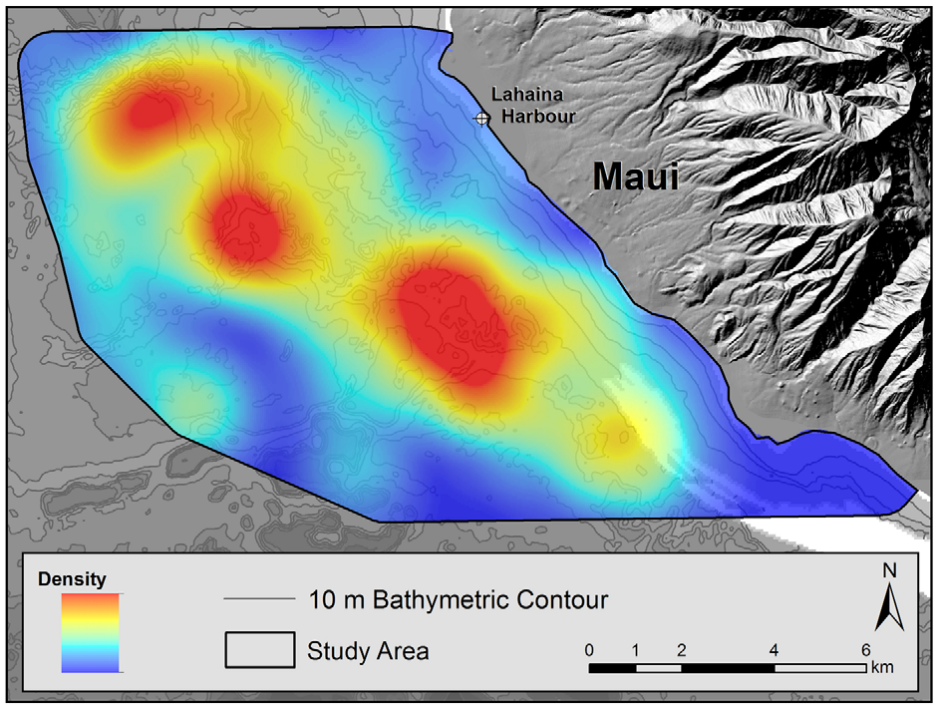
Density of female-calf humpback whale groups in the Au'au Channel, Hawaii, based on systematic surveys conducted during March 2008–2010. This map uses kernel density estimation to create a continuous surface representation of the densities of sightings of female-calf pairs within 1 km^2^ cells (Spatial Analyst ArcView 9.3). Values range from 0 (lowest density – blue) to 1.9 (highest density – red).

To examine female-calf habitat use in relation to habitat availability within the study area, we sub-divided the study area according to four key environmental variables (distance from shore, depth of water, nature of the bottom terrain (terrain was described as rugged or flat) and proximity to the harbor). We compared levels of habitat use by female-calf groups to the proportional availability of each type of habitat within the study area, using Neu's Indices. Habitat use was uneven relative to distance from shore (using Kolmogorov-Smirnov (KS) goodness of fit test for continuous data; *D*_86_ = 0.155, *P* = 0.02), water depth (*D*_86_ = 0.320, *P*<0.001), proximity to the harbor (*D*_86_ = 0.301, *P*<0.001) and in relation to the nature of the terrain (rugged vs. flat terrain; Pearson Chi-Squared test, with Yates Correction; *X*_c_^2^_1, 86_ = 4.702, *P* = 0.025–0.05). Regions where the 95% confidence intervals of observed counts were entirely below expected counts based on habitat availability were classified as avoided areas. For female-calf groups, avoided areas included shallow waters (<20 m), regions both closest to (<2 km) and most removed from (>8 km) the harbor and regions of flat terrain. Regions where 95% confidence intervals of observed counts were entirely above expected counts based on habitat availability were classified as preferred areas. For female-calf groups, preferred areas included water depths between 40–60 m, regions between 4–6 km from the harbor and regions of rugged topography (Table 2, Figure 3).

**Figure 4:**
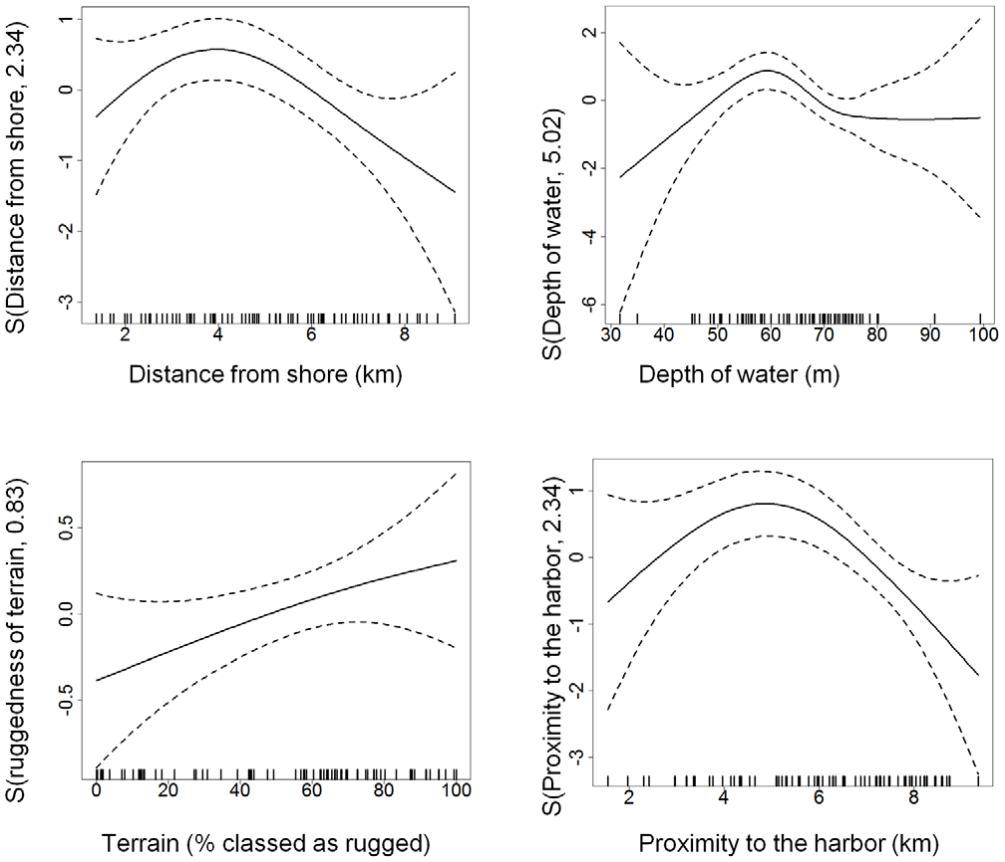
Plots to show the non linear effects of environmental variables on levels of habitat use in female-calf humpback whale groups along the eastern shoreline of the Au'au Channel, Maui, Hawaii.

To evaluate the relative influence of the different environmental factors, we constructed a series of generalized additive models (GAM), using the four environmental variables described (distance from shore, depth of water, nature of terrain and proximity to the harbor) as possible predictors of presence/ absence of female-calf groups within 1 km^2^ grid squares across the study area. The models identified non linear trends in relation to depth, distance from the shore and proximity to the harbor while levels of occurrence increased with increasing percentage of rugged terrain within the grid squares (smoothed curves for each environmental variable are provided in Figure 4). Based on the comparison of AICc (second-order bias correction for Akaike's Information Criteria) values, proximity to the harbor was the single most influential explanatory factor for female-calf occurrence across the study area. However, this explained only a small amount of the total deviance observed (16.4% – see Table 3).

Subsequent construction and evaluation of cumulative models including combinations of the four predictor (environmental) variables and potential interactions between these variables explained considerably more of the variation (up to 44.5%). Based on the comparison of AICc values, several models warranted equal consideration (ΔAICc <2). Of these, the models that explained most deviance included proximity to the harbor, depth of water and terrain. Inclusion of these three individual explanatory variables independently explained 44.3% of the deviance in the model, and adding pair-wise interactions between these three factors further increased the deviance explained by a slight margin, to 44.5% (Table 3).

**Table 2:** Habitat preference in female-calf humpback whale groups along the eastern shoreline of the Au'au Channel, Maui, Hawaii.

Habitat	Area (km^2^)	Proportion of total study area (P_i)_	Actual counts (n of groups)		Observed proportions & 95% CI for counts (Oi)	Inference	Neu's standardized selection index
			Exp.	Obs.			
Distance from shore							
<2 km	30.8	0.247	21.2	9	0.105 (0.027–0.205)	avoided	0.093
2–4km	25.5	0.205	17.6	27	0.313 (0.185–0.443)	neutral	0.304
4–6km	24.9	0.201	17.2	24	0.279 (0.155–0.404)	neutral	0.277
6–8km	23.9	0.192	16.5	17	0.197 (0.087–0.308)	neutral	0.204
>8km	19.4	0.153	13.2	9	0.104 (0.012–0.174)	neutral	0.121
Depth							
<20m	10.2	0.082	7.0	1	0.012 (−0.018–0.041)	avoided	0.035
20–40m	16.6	0.133	11.5	7	0.081 (0.005–0.157)	neutral	0.151
40–60m	35.6	0.285	24.6	37	0.430 (0.293–0.568)	preferred	0.374
60–80m	58.5	0.470	40.5	39	0.453 (0.315–0.592)	neutral	0.239
>80m	3.6	0.029	2.5	2	0.023 (−0.019–0.065)	neutral	0.201
Topography							
Rugged	*61.0	0.506	43.5	54	0.628 (0.511–0.745)	preferred	0.621
Flat	*59.5	0.494	42.5	32	0.372 (0.255–0.489)	avoided	0.378
Proximity to Lahaina Harbor							
<2km	6.5	0.052	4.5	2	0.023 (−0.019–0.065)	neutral	0.094
2–4km	17.9	0.144	12.4	12	0.139 (0.043–0.236)	neutral	0.205
4–6km	26.9	0.216	18.6	32	0.372(0.238–0.506)	preferred	0.364
6–8km	36.4	0.292	25.1	25	0.291(0.165–0.417)	neutral	0.211
>8km	36.8	0.296	25.4	15	0.174(0.069–0.280)	avoided	0.125

* Totals slightly less than entire study region due to slight gaps in bathymetry data (Δ6.5 km^2^). Neu's indices provide standardized estimates of habitat use, based on habitat availability. Regions were classified as preferred (where 95% CI's of observed group counts (Oi) were entirely above the expected counts based on habitat availability (Pi)) and avoided (where 95% CI's of observed counts were entirely below the expected counts). In all other (neutral) regions, 95% CI of observed counts included the expected count.

**Table 3:** AIC values for generalised additive models describing the influence of environmental factors on the occurrence of female-calf humpback whale groups in the Au'au Channel, Maui, Hawaii.

Single explanatory variables	AICc	ΔAICc	Dev Exp.
Female-calf groups			
s(Distance to shore)	99.25	5.71	10.9
s(Depth)	99.20	5.67	16.1
s(Terrain)	104.09	10.56	3.62
s(Proximity to harbor)	93.53	0	16.4
Cumulative models			
s(Proximity to harbor)	93.53	2.38	16.4
s(Proximity to harbor)+ s(Depth)	91.67	0.52	24.1
s(Proximity to harbor)+ s(Depth) +s(Terrain)	91.15	0	44.3
s(Proximity to harbor)+ s(Depth) +s(Terrain) + s(distance to shore)	92.15	1	44.3
s(Proximity to harbor)+ s(Depth) +s(Terrain) + (Proximity to the harbor*Terrain)	91.25	0.1	44.5

Legends:

“s” identifies smoothed data.

“*”denotes terms representing interactions between variables.

AICc – second order Akaike's Inspection Criteria.

ΔAICc – relative change in AIC value, compared to the lowest value recorded.

Dev Exp – provides the estimated percentage of deviation that can be explained by the variable(s) in the model.

To further clarify the nature of any potential interactions between proximity to the harbor, water depth and terrain, we compared levels of habitat use of preferred depth (i.e. 40–60 m) and preferred terrain (i.e. rugged regions) where the preferred region fell within 4 km of the harbor to levels of use of preferred habitat in adjacent areas that lay between 4 and 6 km from the harbor. We used Neu's Indices for these comparisons. For preferred depths (40–60 m), levels of use were not significantly different; (Neu's Indices  = 0.39 within 4 km of the harbor vs. 0.61 between 4 and 6 km; *Xc^2^_,1, 21_* = 1.789, *P*>0.1). For preferred (rugged) terrain, levels of use of rugged terrain within 4 km of the harbor were significantly lower than levels of use of rugged terrain further away (4–6 km) from the harbor, (Neu's Indices  = 0.35 vs. 0.65; *X^2^_1, 37_* = 38.941, *P* <0.001).

### Distribution of adult groups

A total of 62 adult-only groups, (including single animals, pairs of animals and groups of >2 animals) were encountered during the transect survey. For these groups, the mean distance from shore was 5.06 (s.d. 1.95) km, the mean depth of water was 63.3 (s.d. 10.7) m, mean proximity to the small boat harbor (Lahaina Harbor) was 6.26 (s.d. 1.77) km and a slight majority (55%; 34 of 62) of groups were sighted in regions of flat terrain (where bottom topography principally comprised of flat, sandy basins [60]).

Next, we examined levels of habitat use compared to habitat availability for adult-only groups using Neu's Indices. For the adult-only groups, levels of habitat use were uneven for distance from shore (*D*_62_ = 0.161, *P*<0.001), water depth (*D*_62_ = 0.231, *P*<0.001) and proximity to the harbor (*D*_62_ = 0.266, *P*<0.001), however there was no evidence of heterogeneity relative to the nature of the terrain (*X*_c_^2^_1, 62_ = 1.1798, *P*>0.10). Regions that adult-only groups avoided included shallow (<40 m), inshore (<2 km) waters, and regions most removed from (>8 km) the harbor. The only clear preference demonstrated by adult groups was for regions between 6 and 8 km from the harbor. There was also a borderline indication of preference for waters of depths 60–80 m (the lower limit of the 95% confidence interval for the proportion of sightings observed in this region was precisely equivalent to the expected proportion based on habitat availability; Table 4).

Using generalized additive models (GAM) to evaluate the relative influence of the four different environmental factors (depth of water, distance from shore, bottom terrain and proximity to the harbor), results identified the nature of the terrain, closely followed by water depth as the most influential explanatory factors for adult presence/absence, based on the comparison of ΔAICc values, however the most influential single factor (terrain) explained only 11% of the deviance in the model (Table 5). Compiling the explanatory variables into cumulative GAM models produced moderate increases in model performance. The best model selected on the basis of ΔAICc values included all four variables plus the interaction between distance from shore and depth, and explained 30.3% of the deviance in the dataset (Table 5).

**Table 4:** Habitat preference in adult-only humpback whale groups along the eastern shoreline of the Au'au Channel, Maui, Hawaii.

Habitat	Area (km^2^)	Proportion of total study area (P_i)_	Actual counts (n of groups)		Observed proportions & 95% CI for counts (Oi)	Inference	Neu's standardized selection index
			Exp.	Obs.			
Distance from shore							
<2 km	30.8	0.247	15.3	2	0.032 (−0.026–0.090)	avoided	0.026
2–4km	25.5	0.205	12.7	21	0.322 (0.170–0.475)	neutral	0.313
4–6km	24.9	0.201	12.4	14	0.242 (0.102–0.382)	neutral	0.240
6–8km	23.9	0.192	11.9	20	0.322 (0.170–0.475)	neutral	0.334
>8km	19.4	0.153	9.5	5	0.081 (−0.008–0.170)	neutral	0.105
Depth							
<20m	10.2	0.082	5.1	0	0.000 (0.000–0.000)	avoided	0.000
20–40m	16.6	0.133	8.3	1	0.000 (−0.025–0.057)	avoided	0.000
40–60m	35.6	0.285	17.7	22	0.355 (0.198–0.511)	neutral	0.448
60–80m	58.5	0.470	29.2	39	0.629 (0.470–0.787)	neutral*	0.481
>80m	3.6	0.029	1.8	0	0.000 (0.000–0.000)	avoided	0.000
Topography							
Rugged	*61.0	0.506	31.4	28	0.452 (0.310–0.593)	neutral	0.446
Flat	*59.5	0.494	30.6	34	0.548 (0.407–0.690)	neutral	0.556
Proximity to Lahaina Harbor							
<2km	6.5	0.052	3.2	1	0.016 (−0.025–0.057)	neutral	0.071
2–4km	17.9	0.144	8.9	6	0.097 (0.000–0.193)	neutral	0.153
4–6km	26.9	0.216	13.4	19	0.306 (0.156–0.457)	neutral	0.324
6–8km	36.4	0.292	18.1	29	0.468 (0.305–0.631)	preferred	0.365
>8km	36.8	0.296	18.3	7	0.113 (0.009–0.216)	avoided	0.088

* Totals slightly less than entire study region due to slight gaps in bathymetry data (Δ6.5 km^2^). Neu's indices provide standardized estimates of habitat use, based on habitat availability. Regions were classified as preferred (where 95% CI's of observed group counts (Oi) were entirely above the expected counts based on habitat availability (P_i)_) and avoided (where 95% CI's of observed counts were entirely below the expected counts). In all other (neutral) regions, 95% CI of observed counts included the expected count. * borderline – neutral/preferred.

**Table 5:** AIC values for generalised additive models describing the influence of environmental factors on the occurrence of adult humpback whales in the Au'au Channel, Maui, Hawaii.

Single explanatory variables	AICc	ΔAICc	Dev Exp.
Adult-only (singles and groups)			
s(Distance to shore)	104.20	3.24	4.9
s(Depth)	106.66	5.7	10.9
s(Terrain)	100.96	0	11
s(Proximity to harbor)	104.26	3.3	5.9
Cumulative models			
s(Terrain)	100.96	0.62	11
s(Terrain)+ s(Depth)	104.96	4.62	17.1
s(Terrain)+ s(Depth) +s(Proximity to harbor)	105.77	5.43	23.3
s(Terrain)+ s(Depth) +s(Proximity to harbor)+ s(Distance to shore)	101.56	1.22	28.0
s(Terrain)+ s(Depth) +s(Proximity to harbor)+ s(Distance to shore) + (Distance to shore* Depth)	100.34	0	30.3

Legends:

“s” identifies smoothed data.

“*”denotes terms representing interactions between variables.

AICc – second order Akaike's Inspection Criteria.

ΔAICc – relative change in AIC value, compared to the lowest value recorded.

Dev Exp – provides the estimated percentage of deviation that can be explained by the variable(s) in the model.

### Group location according to social group composition

**Figure 5:**
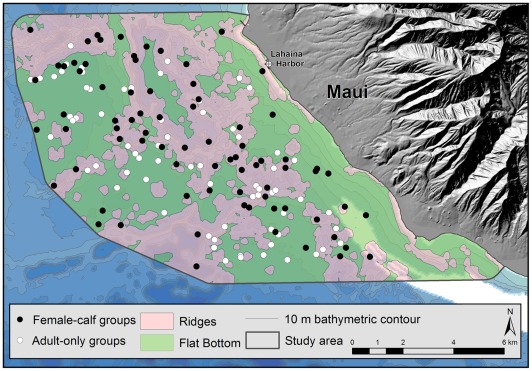
Humpback whale sightings along the eastern shoreline of the Au'au Channel, Maui, Hawaii, classified by presence (female-calf groups) or absence (adult-only groups) of a calf in the group.

Overall, comparing mean locations between female-calf groups and adult-only groups with regards to distance from shore, water depth and proximity to the harbor, differences were not significant (Table 6). A power analysis confirms the reliability of these results ((1-β) = 0.855, G-power [61]). However, relative to bottom topography (rugged vs. flat terrain), when female-calf groups were compared to adult-only groups, female-calf groups were more frequently associated with rugged terrain (Pearson *X^2^_1, 148_* = 4.532, *P* = 0.033; Figure 5).

**Table 6:** Locations of humpback groups classified according to the presence or absence of a calf.

	Female-calf groups	Adult-only groups	*t* value	d.f.	p-value
Distance from shore (km)	4.75 (2.27)	5.06 (1.95)	−0.856	146	0.393
Depth (m)	58.8 (14.7)	63.3 (10.7)	−2.049	139.5	0.042
Distance to the harbor (km)	5.94 (1.99)	6.26 (1.77)	−1.022	146	0.308

Mean (s.d.) values provided.

Differences were not significant, once corrections for multiple testing were taken into consideration (α/k = 0.016).

**Figure 6:**
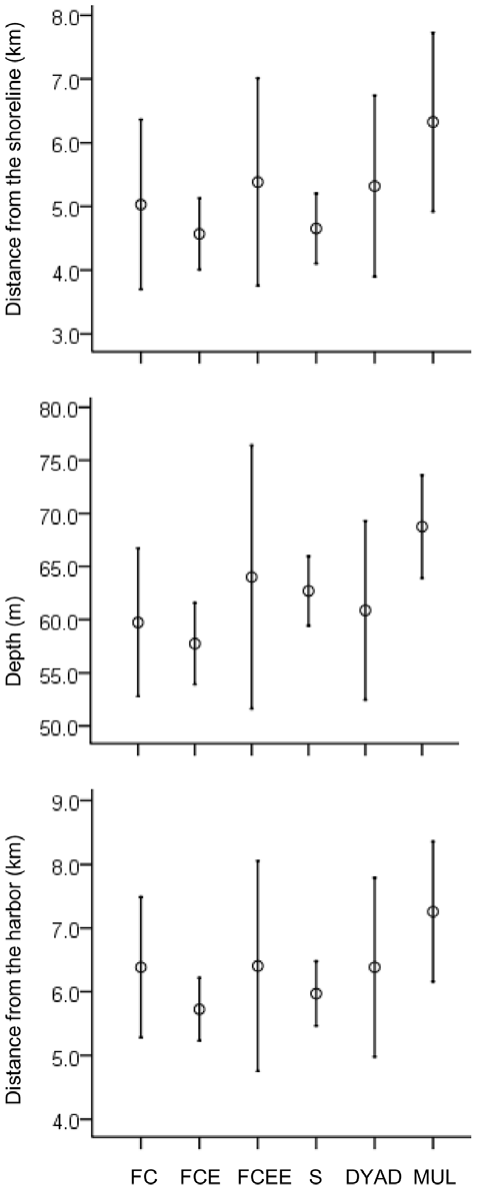
Relative locations of humpback whale groups in the Au'au Channel, classified by precise social group composition. Error bars indicate 95% CI for each mean. Legend: FC – female-calf, FCE – female-calf +escort, FCEE – female-calf +>1 escort, S – single adults, Dyad – adult pairs, MUL – adult groups>2. When groups were classified by precise composition differences in mean distances from shore, depth of water and proximity to the harbor were not significant (for distance from shore, ANOVA; F_5, 142_ = 1.488, p = 0.197, for depth F_5, 142_ = 1.656, p = 0.149 and for distance from the harbor; F_5, 142_ = 1.642, p = 0.182).

Classifying groups by precise social composition of the group (unaccompanied female-calf pairs, female-calf pairs with a single escort, female-calf pairs with >1 escort, single adults, dyad adult pairs, and adult-only groups of >2) also indicated that differences in location were non-significant. As power in this analysis was moderately low ((1-β) = 0.62), we pooled the data into four social group classifications (unaccompanied female-calf pairs, escorted female-calf pairs, single adults and dyad pairs, and adult-only groups of >2). This increased the power ((1-β) = 0.71), but the results remained non-significant, with no variation in location according to depth, distance from shore or proximity to the harbor according to social group (Figure 6). There was no detectable association with terrain according to precise social composition, either using 4 pooled groups (Pearson *X^2^_3, 148_* = 5.071, *P* = 0.167) or when using 6 more precisely defined groups (Pearson *X^2^_5, 148_* = 9.590, *P* = 0.088).

## Discussion

Accurate and up-to-date information on local patterns of habitat use is a pre-requisite for effective conservation of marine fauna, especially in heavily used regions such as coastlines. In this study, based along the coastline of West Maui, Hawaii, preferred female-calf habitat comprised waters between 40–60 m deep and regions that were between 4 and 6 km from Lahaina Harbor. Maternal females (females accompanied by a first season calf) avoided regions that were within 2 km of the coastline and regions where depths were less than 20 m. Notably, female-calf pairs also favored regions of rugged bottom topography and avoided regions where bottom topography was flat.

Generalized additive models (GAM's) using four different environmental variables (distance to shore, depth of water, nature of terrain (classified as rugged or flat) and proximity to the harbor) identified proximity to the harbor as the single most influential factor predicting habitat use by maternal females in this region; regions with highest levels of vessel traffic saw lowest levels of use by maternal females. Additionally, while female-calf groups showed a preference for rugged bottom terrain across the extent of the study area, where regions of rugged terrain fell within close proximity (4 km) of the harbor, these regions were used less than rugged regions further removed from the harbor.

Social segregation or stratification, though typically characteristic of humpback whale breeding grounds, was minimal in this region; we speculate that this lack of clearly defined stratification may be a consequence of the avoidance of coastal waters.

### Maternal habitat use

The avoidance of coastal waters by maternal females in this region stands in stark contrast to trends seen elsewhere. While high levels of boat traffic in coastal regions provide one possible explanation for these low levels of use, other explanations should be considered. For example, these areas could be beyond the typical range of tolerance for female-calf pairs (i.e. these areas could be too shallow or too close to the shoreline). Reviewing habitat use in maternal females across other regions suggests that this is not the case. With regards to depth, maternal females favor equally shallow water depths (of less than 20 m) on several key humpback whale breeding grounds (e.g., [28], [62], [63]). Looking across the range of different breeding regions surveyed to date, female-calf groups consistently favor waters less than 50 m deep [32], [64]–[66]. In comparison, maternal depth preference across different regions within Hawaii is variable. On the Penguin Banks, an offshore region that ranks a close second to the Au'au Channel as a favored female-calf region within Hawaii [16], female-calf groups congregate on the south-western tip of the bank, in the shallowest waters available where depths range between 30–40 m deep [16]. In contrast, along the Kohala coastline of the Island of Hawaii and the north shore of Kauai, regions that are also lightly used by female-calf pairs [16], maternal females use comparatively deeper water than in other regions (mean reported depths range from 56 to 83 m respectively [54], [55]). In this study, as the mean depth for female-calf pairs was recorded as 58 (s.d.14.89) m, evidently female-calf pairs in the Au'au Channel are using deeper waters compared to both the Penguin Banks and the Kohala coastline.

With regards to proximity to the shoreline, on alternate breeding grounds maternal females are routinely found within 1–2 km of the shoreline. In many of these areas, steep shoreline gradients place female-calf pairs close to the coastline in the shallowest water available. Examples include Antongil Bay, Madagascar, [28], the coastline of the Osa Peninsula, Costa Rica [64], the Kohola coastline of the Island of Hawaii, Hawaii [54] and the North shore of Kauai, Hawaii [55]. In contrast, where shallow waters extend offshore, such as on the Abrolhos Bank in Brazil and in coastal regions of Ecuador, female-calf groups favor areas up to 10 km from the shoreline [62], [63], while on the Penguin Banks in Hawaii, the choice of the shallowest waters on the banks places female-calf pairs some 40 km off the nearest coastline. Taken cumulatively, these examples suggest that proximity to the shoreline may be a flexible feature in maternal breeding habitat choice. In contrast, maternal preference for the shallowest water available is a consistent trait. Habitat use by maternal females in the Au'au Channel appears to be the exception; here, maternal females preferentially use deeper waters that lie further offshore and avoid shallow, shoreline areas.

Given the correlation between high levels of vessel traffic in shoreline waters and the avoidance of these regions by female-calf pairs, it would be easy to assume that this is a causative relationship. Numerous examples of animals altering their patterns of habitat use or avoiding previously favored habitat in response to increasing traffic or vessel activity have been reported elsewhere. Examples include both terrestrial (e.g., [67]–[70] and marine systems (e.g., [36], [71]–[73]), with maternal females frequently exhibiting high sensitivity to these disturbances (e.g., [74]–[76]). In this study, we see the correlation of the effects of shoreline and harbor proximity; overall, the lowest levels of maternal habitat use (indicated by the Neu's Indices) are seen in areas closest to each of these features, where levels of vessel traffic are highest. Additionally, evidence from the GAMs constructed identifies harbor proximity as the single most influential factor in maternal habitat choice. Still, care should be taken in attributing any influence on maternal habitat use entirely to the presence of vessel traffic. Many other issues, such as changes in water quality, increasing run-off associated with changing patterns of land-use and changes in the acoustic environment may also impact these regions and warrant consideration. Further research is required to determine the degree to which any of these other factors may or may not be involved.

### Is this a long term trend?

Comparing the results of this study to previous work on female-calf use of coastal waters in this area suggests that an increasing distance from the shoreline could potentially be the continuation of a long term trend. During the early eighties, Glockner-Ferrari and Ferrari [50], [51] reported a drop in the proportion of female-calf pairs sighted within 0.4 km of the shoreline, from 36.4% of all female-calf groups sighted in 1981 to 17.2% of all female-calf groups sighted in 1983. Salden [52] also reported an increase in the mean distance from shore for female-calf pairs between 1981 and 1986; the mean distance from shore for female-calf pairs was reported as between 3–4 km by the end of this study. Certainly, the methods used vary both between these two early studies, and between these early studies and the current study. Notwithstanding, within this data set only 1 of 86 female-calf groups was sighted within 0.4 km of the shoreline (this represents 1.2% of groups) and while the study area defined in this study was similar in dimensions to Salden's study area [52], the mean distance from shore for female-calf pairs in this study was 4.75 (s.d. 2.27) km. These comparisons certainly suggest that levels of use of shoreline regions by maternal females have decreased between the early seventies and today, with the caveat that some of the variability between the data sets may reflect different survey techniques. Further research using consistent survey methods is warranted to determine whether or not this is a long term trend. In the meantime, this study demonstrates that female-calf pairs currently exhibit a clear preference for mid-channel over coastal waters.

### Maternal trade-offs in habitat use

For maternal females, coastal waters provide a range of potential benefits, ranging from reduced costs of swimming for young animals [34] to reduced predation pressure [77], [78]. However, where favored regions comprise exposed offshore banks, such as the Penguin Banks, protection is minimal and waters are frequently rough, and where breeding grounds lie in warmer water, predation pressure is minimal [20], [79]. An alternate explanation for the use of shallow water is as a means of facilitating social stratification and thereby reducing male harassment. This has been documented in multiple alternate regions across a wide range of mysticete breeding grounds (e.g., [28], [30], [78]) and in some regions, segregation of female-calf pairs from actively breeding adults within wintering grounds is a deterministic factor in terms of calf survival [27].

Evidence from maternal regions within Hawaii confirms that a degree of social segregation is in place in some Hawaiian waters. Female-calf pairs cluster in the shallowest water on the Penguin Banks while adult-only groups use deeper waters across the extent of the banks [16] and along the Kohala shoreline of the Island of Hawaii, female-calf groups come closer to shore and use shallower depths than adult groups [16], [54]. In our study area, while we saw no difference in mean depth or distance from shore for female-calf groups vs. adult-only groups, we did detect evidence of slight separation between the two groups using Neu's Indices, where habitat use is standardized against habitat availability. Adult groups exhibited a borderline preference for 60–80 m depths, while female-calf preference was for depths between 40–60 m. However for adult groups, the 40–60 m region came a close second in terms of levels of use (Neu's Indices  = 0.448 for 40–60 m and 0.481 for 60–80 m) and similarly for female-calf groups, levels of use of the 60–80 m depths were only slightly lower than levels of use for the 40–60 m region (Neu's Indices  = 0.374 for 40–60 m and 0.239 for 60–80 m). Cumulatively, this suggests some overlap, with both groups in fact using depths within 40–80 m range.

A lack of clearly defined separation in mid–channel waters would carry potential costs for female-calf pairs, should this increase the likelihood of female-calf associations with multiple male groups. Recently conducted play back experiments conducted in this area document female-calf groups moving away from the recorded sounds of multiple male groups [80] and previous research has demonstrated that when associated with multiple male groups, female-calf energy costs rise by around 30%, as pairs switch from rest to travel and swimming speeds increase [35].

The costs of persistence in highly trafficked regions for female-calf humpback whale pairs are also well-documented. Female-calf groups are more sensitive to vessel presence compared to other social groups [81], and in Hawaiian waters, calves are more prone to vessel strikes than other age cohorts [57]. Maintaining a startle response to vessel traffic reduces the likelihood of injury, but increases stress [45], [48]. Additionally, female-calf pairs communicate acoustically [82], [83], and this also could be impacted in highly trafficked waters [84].

Evidently, for maternal females both inshore and mid-channel waters within the Au'au Channel may be associated with a range of potential costs and benefits. While the use of shallow waters is typically seen as a method of reducing the likelihood of associations with multiple male groups, female-calf pairs along the West Maui shoreline and close to the harbor will be exposed to higher levels of vessel traffic. Mid-channel waters reduce exposure to vessel traffic, but place female-calf pairs in closer proximity to multiple male groups. In the Au'au Channel, as maternal females avoid coastal waters in favor of mid–channel regions, this would suggest a potential trade-off between the costs and benefits of these different areas. As our results indicate maternal females favor deeper waters over shallow, coastline areas along the West Maui shoreline, this trade-off plays out in favor of maternal use of mid-channel waters. Several other studies documenting maternal response to human disturbances provide illustrations of increased maternal sensitivity to disturbance resulting in similar trade-offs in habitat choice (e.g., [85], [86]).

### Single escorts and the bodyguard hypothesis

Pertinent to this potential trade-off may be the predominance of female-calf-single escort groups in this area. The majority of maternal females in the Au'au Channel (70–85%) are escorted by a single male whale [87]. Single escort associations may alter the behavioral budget of the female-calf pair, increasing energetic consumption to some extent [35], [80], but these single males also fit Mesnick's ”body guard” definition [35], [88], [89], potentially deflecting male aggression and reducing the likelihood of attracting multiple males [90], [91]. On other humpback whale breeding grounds where social segregation is more distinctly defined, the frequency of female-calf–single escort groups is much lower than in the Au'au Channel (e.g., Samana Bay, Dominican Republic, 35% [32], coastal Ecuador; 23.5% [63] Albrohos Bank, Brazil; 41% [92]). Meanwhile, the incidence of maternal associations with multiple male groups on these stratified breeding grounds (Samana Bay, Dominican Republic; 6% [32], coastal Ecuador; 9.5% [63] Albrohos Bank, Brazil; 6.5% [92]) is only slightly higher than seen in this area (10.5% in this study, which concurs with estimates from other studies [35] [54]). Potentially, as maternal females disperse from the coastal waters of the Au'au Channel, associations with a single male escort may comprise a behavioral mechanism that offsets the risk of increased harassment by proximate multiple male groups in mid-channel waters. Similar behavioral mechanisms have been reported elsewhere. For example, in an evolutionarily comparable species, Grevy's zebra (*Equus grevyi*), associations between lactating females and single males serve as an effective mechanism to reduce male harassment of the female in regions where the preferred habitat of lactating females and breeding males overlap [93].

### Bottom topography and habitat use in maternal females

One of the more surprising results in this study was the noted preference in maternal females for regions of rugged bottom topography. Topography has been cited as a determinant factor in one other study of female-calf habitat use; however, preference was seen for regions of gentle slope [64]. A simple explanation may be that the rugged regions provide an alternate shallow water environment for maternal females. The mid-channel regions where bottom terrain is rugged essentially comprise ridges of drowned karst reef, and these regions are slightly shallower than the surrounding flat, sandy basins that comprise the flat terrain [60]. As the regions of rugged and flat terrain are interspersed across the channel, maternal preference for these regions would be not expressed in distance from shore, but as these regions are slightly shallower, it may be expressed in depth preference. Tenuous details may also be drawn together to suggest a connection between bottom topography, social role and reproductive strategies in humpback whales. Primarily, these relate to site choice; and more specifically, the preference and suitability of flat sandy basins for the broadcast of humpback whale song [34]. In this study, we did detect a slight preference in singing whales and dyad pairs for sandy, flat terrain; however, as the role of song remains in debate, our results at most suggest a novel route for further research.

### Timing of the study

This study was restricted to the latter portion of the season and the implications of this should be considered, as they could provide alternate explanations for the patterns of association and habitat choice that we report here. For example, during this portion of the season, maternal females could relocate from coastal to mid-channel waters in search of post-partum mating opportunities. Reviewing the accumulation of evidence would suggest that this is unlikely. Firstly, necropsy data indicates that only 8.5% of maternal females ovulate postpartum [24], the mean birthing interval for humpback whales is estimated as 2.38 years [94], and annual birthing intervals, though recorded on rare occasions [51], most likely constitute less than 2% of all calving intervals [95]. Moreover, by the final stages of the breeding season, an existing calf represents a substantial energetic investment for a maternal female [25]. Therefore, we speculate that it would be highly unusual for a female with an existing, healthy calf to solicit mating opportunities and augment the high costs of lactation with costs of concurrent gestation [25]. Finally, recent playback experiments in this region document maternal females consistently moving away from the sounds of surface active males [80]. Taken cumulatively, these findings support the assumption that maternal females do not typically seek out or solicit male attention or mating opportunities, at this or any other time in the season.

Alternatively, males could relocate from deeper regions to mid channel waters during this portion of the season, and effectively swamp previously well-defined patterns of stratification. Most non–maternal females will have already left the breeding grounds [96], and maternal females attract more male attention as the season progresses [87]. However, given the negative impacts of multiple male attention [27], [35], any relocation or increase in males in near-shore waters would be expected to amplify maternal avoidance strategies. Thus, the continued avoidance of coastal waters during this portion of the season underscores the reduced appeal of these areas for maternal females in this region.

### In conclusion

Reviewing maternal habitat choice across Hawaiian waters suggests a flexible response to changing environmental conditions. Tagging and photo-id studies confirm that maternal females in Hawaiian waters move between favored female-calf regions both within and between seasons [17], [97], [98]. In regions where shoreline waters are available to them and provide shallower water than surrounding areas, maternal females adopt the typical patterns of social stratification that characterize mysticete breeding regions [54], [55]. However, where the shallower waters lie offshore, or where the available shallow waters carry other costs, such as increased human disturbance, the results of this study support the conclusion that maternal females forego the protection of the shoreline; they favor alternate offshore waters, and may adopt alternate behavioral strategies to offset the potential costs of these regions.

Within Hawaii, the Au'au Channel provides the most extensive expanse of protected, shallow water within the island chain and at this point, most recent audits indicate that maternal females maintain a significant preference for this region [16], [17]. Further research is required to fully understand the appeal of this region for maternal females. Our results indicate that cumulatively, the factors considered here account for almost 50% of the variability in maternal habitat choice; therefore other factors, potentially ranging from alternate environmental factors to social dynamics, may yet prove to play a key role in directing fine-scale habitat use in maternal females in this area. For now, these results highlight the key areas currently used by maternal females during the latter portion of the season, and as this represents a crucial pre-migratory growth period for calves [59], we would suggest that these areas warrant targeted, careful and precautionary management.

## Methods

### Ethics Statement

The study was conducted under NMFS scientific research permit # 10018 and under associated Hawaii State permits, SH2008-, SH2009- and SH2010-08. Full details of the precise research protocols used in this study were carefully reviewed by the Office of Protected Species, prior to issuance of the above research permit. Inherent in this review, is the requirement that every effort be made to minimize any impact on animals during research activities. As this detailed and extensive review had been conducted by experts in this field, further ethical review by the co-operating institution, California State University Channel Islands was not required. All research protocols additionally comply with the Endangered Species Act (1973) and the Marine Mammal Protection Act (1972) (https://apps.nmfs.noaa.gov/docs_cfm/laws_and_regulations.cfm).

### Study Site

Surveys were conducted in March 2008, 2009 and 2010 along the eastern shoreline of the Au'au Channel, West Maui (∼20^°^ 52′ N, 156^°^ 40′ W). The Au'au Channel is essentially a drowned land bridge that once connected the islands of Maui and Lanai. It features gently sloping shoreline gradients, maximum water depths of ∼ 150 m, a median depth of 55 m and complex mid channel topography that includes sea mounts and ridgelines, interspersed between steep-sided sandy basins [60]. Our study area (Figure 1) extended from the Maui shoreline to either the mid– or deepest point of the channel at each minute of latitude, whichever lay furthest offshore. Northern and southern limits were set within the lee provided by the West Maui Mountains, thereby minimizing local variations in sightability and sea state across the study area and ensuring that the small boat harbor in Lahaina, West Maui was included.

### Survey Design

We constructed an equally spaced zigzag sampling transect between parallel waypoints at 1 minute of latitude intervals across the study area (Figure 1); this ensures equal probability of coverage across the site, with completed transects comprising independent samples [99]. Daily starting points were chosen randomly, all surveys were conducted in sea states of <Beaufort 2 and 2 different survey vessels (a 6 m and an 8 m powerboat) traveled at approximately 9 km hr^−1^ (5 knots) along the survey lines. Two designated naked-eye observers scanned on opposite sides of the vessel and any sightings within 90 degrees on either side of the forward bow and within an estimated 1 km to either side of the survey line were recorded. Effective strip width for humpback whales on boat based surveys in Californian waters with a set speed of 5 knots (9.4 km hr^−1^) has been previously estimated as 3.2 km [100]; consequently we assume that sightability within 1 km of a transect line within Hawaiian waters, for surveys conducted in Beaufort sea scale <2, would be 100%. Detection probability, based on the ratio of surface to submerged time, may vary with the social composition [15]; based on a boat speed of 9.4 km hr^−1^, mean detection probability was slightly higher for groups containing calves vs. adult-only groups (0.36 vs. 0.31).

Locations of sighted whales were recorded as latitude and longitude on handheld GPS units after the whales(s) left the surface. Generally, as humpback whales dive they leave a footprint, (a vortex of flattened water) that persists at the surface, so wherever possible, this was used as a marker. Otherwise, location was estimated based on the last sighting of the first surface interval observed. Group composition was established following protocols described in Cartwright and Sullivan, [59]. When single adult whales were encountered, a hydrophone (Cetacean Research, Washington, USA: Model CR1) was lowered to detect singing, and when loud, potentially local singing was heard, observations were prolonged until the whale sighted surfaced again. Where cessation and subsequent resumption of the song consistently co-incided (over >2 surfacings) with surface observations of the individual, the adult was recorded as a singer. Fluke photo ID's [101] and surface images documenting body markings, lesions and other scars were compiled for all sighted animals and used post-hoc to eliminate any chance of pseudo replication over the course of the day, between survey vessels or within regions of over-lap at the beginning or the end of any successive transects.

### Vessel distribution

Vessel distribution within the study area was monitored over a 10-day period in March 2010. The aim of this short term survey was to provide a representative snapshot of typical trends in vessel traffic levels at the time of year when the study was conducted annually. Numbers of commercial vessels in the region remain relatively constant year-to-year due to slip and launching permit restrictions. Currently, there are 38 commercial permits available for the small boat harbor in Lahaina, 29 for Maalaea Harbor, a small boat harbor approximately 12 km to the south of the study area, and another 6 launching permits allow commercial operators to launch vessels from Mala Wharf, which is a small boat ramp within the northern portion of the study area. Local harbor officials confirmed that all commercial permits were in use during each whale season over the course of the study and financial incentives ensure that commercial operators typically run the maximum feasible number of trips per day. We reviewed their schedules to verify trip frequency, and saw no unusual variations in the numbers of daily trips scheduled over the course of the study. Berthing limitations constrain the numbers of private vessels that operate in this region. The proportion of private vessels and any potential variation in their numbers was assessed during vessel data analysis.

Vessel scans were conducted from a land-based station overlooking the study area, with a maximum of 3 scans daily, and a minimum 3 hr interval between scans. Commercial vessels were typically easily recognizable, so vessels could be identified as commercial or private, and vessel locations were recorded using a surveying theodolite (Sokkia DT520A), with theodolite readings subsequently converted to latitude and longitude in digital degrees using the *Pythagorus* program [102]. Vessel activity was classified as (1) whale-watching, where vessels were within 500 m of a group of whales, traveling intermittently and at approximately the same speed and heading as the whales, (2) transiting, when vessels were underway and travel was primarily uni-directional, and (3) stationary, when vessels were not moving and there was no evidence of active relocation during the period of observation. Vessels on permanent moorings around the harbor were documented but were not included in the scan counts.

### Spatial analysis

A Geographic Information System model (GIS) was constructed using ArcGIS 9.3 (Environmental Systems Research Institute). All whale and vessel sightings were plotted, island profiles were obtained from Digital Elevation Maps from the United States Geological Survey, coastline data came from the Hawaii Data Clearinghouse and water depth was obtained from the Main Hawaiian Islands Multibeam Synthesis web site, (http://www.soest.hawaii.edu/HMRG/Multibeam/index.php) and incorporated as a 50 m bathymetric grid. NOAA's Benthic Terrain Modeler [103] was used to classify areas of complex topography as peaks, crests and depressions, based on fluctuations in gradients. A 750 m buffer constructed around the survey line provided coverage of 86% of the study area without overlap between mid-sections of adjacent transects. As sightings within an estimated 1 km had originally been recorded, this reduced any potential edge effect. Sightings that fell beyond the buffer were discarded, as were sightings from incomplete transects. Although this did reduce the sample size slightly, Strindberg and Buckland [99] advocate these steps as a method of maintaining equal probability coverage across the survey area.

The Spatial Analyst extension of ArcView 9.3, 2010, ET GeoWizards 10.2 [104], and Hawth's Analysis Tools [105] were used to further explore the spatial dimensions of the GIS model. Contours were constructed across the study area at 2 km intervals from the shoreline and 20 m intervals in depth, providing distinct regions classified by distance from shore and water depth. Areas of complex terrain (peaks, crests and depressions) were enclosed within a 100 m buffer to incorporate transitional areas and joined into a single component layer, describing “rugged” topography within the study area; areas outside these regions were annotated as “flat” and co-incided closely with the sandy basins described by Grigg et al. [60]. Proximity to Lahaina harbor was delineated by 2 km-wide, concentric bands centered on the harbor entrance. Estimates of distances to the closest coastline, water depth, nature of the terrain and proximity to the harbor were obtained for all whale locations, areas of the different defined zones were calculated in km^2^ and counts of whale and vessel occurrences within these zones were compiled. For the purposes of statistical modeling, a 1 km square grid overlaying the study area was created and for all grid squares that fell completely within the 750 m buffers of the transect lines, whale occurrence (as presence/absence), median depth, centroid distances to the nearest shoreline and to the small boat harbor, and percentage of rugged topography were compiled. Finally, the distribution of female-calf groups across the study area was summarized in a kernel density map (Spatial Analysis toolbox, ArcView 9.3), using 1 km^2^ cells. This tool essentially compiles density of point locations (female-calf groups) into a smooth, continuous two dimensional layer (Figure 2).

### Statistical analysis

Statistical analyses were conducted using PASW version 18, and R version 2.11.1 (The R Foundation for Statistical Computing, 2006). An ANOVA analysis was used to detect any overall inter-annual variability in mean distances from shore, depths and proximity to the harbor and a test for evidence of association was used to examine variation in choice of terrain between years.

### Assessing habitat use

Neu's Method, with modifications as advocated by McClean et al. [106] and Redfern et al. [107], was used to quantify levels of habitat use relative to availability, for regions defined by distance from shore, depth, nature of terrain (rugged vs. flat) and proximity to the harbor. To detect heterogeneity in habitat use we used the Kolmogorov-Smirnov goodness-of-fit test for continuous data [107], [108] and Pearson's Chi squared test where habitat was classified into discrete categories, with Yates correction for continuity incorporated where the number of groups was only 2 (*ν* = 1). As a follow-up, 95% confidence intervals around proportional use estimates were compared to expected use estimates based on habitat availability, to identify where habitats were selected disproportionately to their availability [109]. Habitat was then designated as (1) avoided, (95% confidence interval (CI) of the observed proportion of sightings in each region was entirely below the expected proportion of sightings), (2) preferred, (95% CI of the observed proportion of sightings in each region was entirely above the expected proportion of sightings) or (3) neutral (95% CI for the observed counts contained the expected proportion). Neu's standardized selection indices were also calculated; these provide directly comparable indices of habitat use. Initially we assessed variations in levels of habitat use for female–calf pairs (female-calf groups) and then repeated the process using adult-only groups.

### Habitat modeling

In order to determine the relative influence of a range of environmental factors, (distance from shore, water depth, nature of terrain, and proximity to Lahaina Harbor) on the distribution of whales within the study area grid, we constructed a series of generalized additive models (GAM; [110]), using the “mgcv” package for R [111]. The environmental factors were considered as potential explanatory variables and the presence/ absence of whales comprised the response variable. Each of the environmental variables was used separately to provide a series of non-linear models reflecting the influence of each individual variable [112]. We applied a binomial model with a clog-log link, which compensated for the inequalities in the frequencies of different values in the binomial response variable that were present in the data. Thin plate penalized regression splines were used (this is the default setting in “mgcv”) and the appropriate degree of smoothing for each curve was assigned by “mgcv”, with a maximum value of *k* = 10. Model selection was based on comparisons of second order Akaike's Inspection Criteria (AICc; [113]) to account for any effect of a small sample size relative to the number of environmental factors considered. Comparisons of AICc values provide a simple, effective, and objective means for model selection. Models with lower AICc values are assumed to best fit the data with the least possible number of parameters. Models with AICc values differing by less than 2 are considered to be equivalent [114]. For model validation we used the gam.check function in “mgcv” to plot residuals, identify any overly influential data points and confirm homogeneity across the data set.

To investigate interactions between variables and improve the explanatory performance of the model, we then used a forwards stepwise procedure to construct multivariate GAMs. We started with the single best performing explanatory variable and added additional variables and then their interactions with the existing variables in the model, based on the improvement of the model's performance. Co-linearity was apparent between most of the explanatory variables (Spearman rho values were between 0.5 and 0.8 for pair-wise comparisons of distance to shore, depth, and proximity to the harbor), however GAM's are resilient to this [114]. Model performance was assessed according to the model's AICc scores and for those where ΔAICc values were with a range of 2, we considered the best model to be the one where the greatest amount of deviance was explained. Models were constructed for female-calf groups and subsequently for adult-only groups.

### Evidence of social stratification

Groups were initially classified based on the inclusion of a calf in the group and subsequently by precise social composition, then differences in mean distance from shore, depth proximity to the harbor and choice of terrain were examined, using standard parametric tests for normal data and non parametric tests for non normal data. The level of significance was set at 0.05, with corrections for multiple testing incorporated as appropriate.

## References

[1] Bossart GD (2006). Marine mammals as sentinel species for oceans and human health.. Oceanography.

[2] Moore SE (2008). Marine mammals as ecosystem sentinels.. Journal of Mammalogy.

[3] Nicol S, Bowie A, Jarman S, Lannuzel D, Meiners KM (2010). Southern Ocean iron fertilization by baleen whales and Antarctic krill.. Fish and Fisheries.

[4] Roman J, McCarthy JJ (2010). The Whale Pump: Marine Mammals Enhance Primary Productivity in a Coastal Basin.. PLoS One.

[5] Williams R, Lusseau D, Hammond PS (2009). The role of social aggregations and protected areas in killer whale conservation: the mixed blessing of critical habitat.. Biological Conservation.

[6] Thompson MJ, Henderson RE (1998). Elk habituation as a credibility challenge for wildlife professionals.. Wildlife Society Bulletin.

[7] Rubin ES, Boyce WM, Stermer CJ, Torres SG (2002). Bighorn sheep habitat use and selection near an urban environment.. Biological Conservation.

[8] Gavin SD, Komers PE (2006). Do pronghorn (Antilocapra americana) perceive roads as a predation risk?. Canadian Journal of Zoology.

[9] Buij R, McShea WJ, Campbell P, Lee ME, Dallmeier F (2007). Patch-occupancy models indicate human activity as major determinant of forest elephant Loxodonta cyclotis seasonal distribution in an industrial corridor in Gabon.. Biological Conservation.

[10] French SS, Gonzalez-Suarez M, Young JK, Durham S, Gerber LR (2011). Human Disturbance Influences Reproductive Success and Growth Rate in California Sea Lions (Zalophus californianus).. PLoS One.

[11] Forney KA (2000). Environmental models of cetacean abundance: reducing uncertainty in population trends.. Conservation Biology.

[12] Harwood J (2001). Marine mammals and their environment in the twenty-first century.. Journal of Mammalogy.

[13] Lusseau D, Bejder L (2007). The long-term consequences of short-term responses to disturbance experiences from whalewatching impact assessment.. International Journal of Comparative Psychology.

[14] Calambokidis J, Falcone EA, Quinn TJ, Burdin AM, Clapham PJ (2008). SPLASH: Structure of populations, levels of abundance and status of humpback whales in the North Pacific..

[15] Mobley JM, Spitz S, Grotefendt R, Forestell P, Frankel A (2001). Abundance of humpback whales in Hawaiian waters: Results of 1993-2000 aerial surveys.. Report to the Hawaiian Islands Humpback Whale National Marine Sanctuary.

[16] Mobley JR, Bauer GB, Herman LM (1999). Changes over a ten-year interval in the distribution and relative abundance of humpback whales (Megaptera novaeangliae) wintering in Hawaiian waters.. Aquatic Mammals.

[17] Craig AS, Herman LM (2000). Habitat preferences of female humpback whales Megaptera novaeangliae in the Hawaiian Islands are associated with reproductive status.. Marine Ecology Progress Series.

[18] Office of National Marine Sanctuaries. (2010). Hawaiian Islands Humpback Whale National Marine Sanctuary Condition Report 2010.. U.S. Department of Commerce, National Oceanic and Atmospheric Administration, Office of National Marine Sanctuaries, Silver Spring, MD,.

[19] Barlow J, Calambokidis J, Falcone EA, Baker CS, Burdin AM (2011). Humpback whale abundance in the North Pacific estimated by photographic capture recapture with bias correction from simulation studies.. Marine Mammal Science.

[20] Corkeron PJ, Connor RC (1999). Why do baleen whales migrate?. Marine Mammal Science.

[21] Ford JKB, Reeves RR (2008). Fight or flight: antipredator strategies of baleen whales.. Mammal Review.

[22] Mehta AV, Allen JM, Constantine R, Garrigue C, Jann B (2007). Baleen whales are not important as prey for killer whales Orcinus orca in high-latitude regions.. Marine Ecology Progress Series.

[23] Forney KA, Wade P, Estes J (2007). Worldwide distribution and abundance of killer whales.. Whales, whaling and ocean ecosystems: University of California Press.

[24] Chittleborough R (1958). The breeding cycle of the female humpback whale, Megaptera nodosa (Bonnaterre).. Australian Journal of Marine and Freshwater Research.

[25] Lockyer C (2007). All creatures great and smaller: a study in cetacean life history energetics.. Journal of the Marine Biological Association of the United Kingdom.

[26] Geist V, Thomas JW (1982). Adaptive behavioral strategies.. Elk of North America ecology and management.

[27] Elwen S, Best P (2004). Female southern right whales Eubalanena australis: Are there reproductive benefits associated with their coastal distribution off South Africa?. Marine Ecology Progress Series.

[28] Ersts PJ, Rosenbaum HC (2003). Habitat preference reflects social organization of humpback whales (Megaptera novaeangliae) on a wintering ground.. Journal of Zoology (London).

[29] Felix F, Haase B (2005). Distribution of humpback whales along the coast of Ecuador and management implications.. Journal of Cetacean Research and Management.

[30] Jones ML, Swartz SL, Jones ML, Swartz SL, Leatherwood S (1984). Demography and phenology of gray whales and evaluation of whale-watching activities in Laguna San Ignacio, Baja California Sur, Mexico.. The Gray Whale.

[31] Mattila DK, Clapham PJ, Katona SK, Stone GS (1989). Population composition of humpback whales, Megaptera novaeangliae, on Silver Bank, Atlantic Ocean 1984.. Canadian Journal of Zoology.

[32] Mattila DK, Clapham PJ, Vasquez O, Bowman RS (1994). Occurrence, population composition, and habitat use of humpback whales in Samana Bay, Dominican Republic.. Canadian Journal of Zoology.

[33] Taber S, Thomas P (1982). Calf development and mother-calf spatial relationships in southern right whales.. Animal Behaviour.

[34] Whitehead H, Moore MJ (1982). Distribution and movements of West Indian humpback whales in winter.. Canadian Journal of Zoology.

[35] Cartwright R, Sullivan M (2009). Associations with multiple male groups increase the energy expenditure of humpback whale (Megaptera novaeangliae) female and calf pairs on the breeding grounds.. Behaviour.

[36] Bejder L, Samuels AMY, Whitehead HAL, Gales N, Mann J (2006). Decline in Relative Abundance of Bottlenose Dolphins Exposed to Long Term Disturbance.. Conservation Biology.

[37] Lusseau D, Slooten L, Currey RJC (2006). Unsustainable dolphin-watching tourism in Fiordland, New Zealand.. Tourism in Marine Environments.

[38] Scheidat M, Castro C, Gonzalez J, Williams R (2004). Behavioural responses of, humpback whales (Megaptera novaeangliae) to whalewatching boats near Isla de la Plata, Machalilla National Park, Ecuador.. Journal of Cetacean Research and Management.

[39] Corkeron PJ (1995). Humpback whales (Megaptera novaeangliae) in Hervey Bay, Queensland: behaviour and responses to whale-watching vessels.. Canadian Journal of Zoology.

[40] Findley LT, Vidal O (2002). Gray whale (Eschrichtius robustus) at calving sites in the Gulf of California, Mexico.. Journal of Cetacean Research and Management.

[41] Rowntree VJ, McGuinness P, Marshall K, Payne R, Sironi M (1998). Increased harassment of right whales (Eubalaena australis) by kelp gulls (Larus dominicanus) at Peninsula Valdes, Argentina.. Marine Mammal Science.

[42] Corkeron PJ (2004). Whale watching, iconography, and marine conservation.. Conservation Biology.

[43] Arnould J, Luque S, Guinet C, Costa D, Kingston J (2003). The comparative energetics and growth strategies of sympatric Antarctic and subantarctic fur seal pups at Iles Crozet.. Journal of Experimental Biology.

[44] Pack AA, Herman LM, Spitz SS, Hakala S, Deakos MH (2009). Male humpback whales in the Hawaiian breeding grounds preferentially associate with larger females.. Animal Behaviour.

[45] Muller MN, Kahlenberg SM, Thompson ME, Wrangham RW (2007). Male coercion and the costs of promiscuous mating for female chimpanzees.. Proceedings of the Royal Society Biological Sciences Series B.

[46] Reid JM, Bignal EM, Bignal S, McCracken DI, Monaghan P (2006). Spatial variation in demography and population growth rate: the importance of natal location.. Journal of Animal Ecology.

[47] Van De POL, Bruinzeel LW, Heg D, Van Der Jeugd HP, Verhulst S (2006). A silver spoon for a golden future: long term effects of natal origin on fitness prospects of oystercatchers (Haematopus ostralegus).. Journal of Animal Ecology.

[48] Walker BG, Boersma P, Wingfield JC (2005). Physiological and behavioral differences in Magellanic penguin chicks in undisturbed and tourist visited locations of a colony.. Conservation Biology.

[49] Herman LM, Forestell PH, Antinoja RC (1980). The 1976/77 migration of humpback whales into Hawaiian waters: Composite description. Marine Mammal Commission, Washington, DC.. Report MMC-77/.

[50] Glockner-Ferrari D, Ferrari M (1985). Individual identification, behavior, reproduction, and distribution of humpback whales, Megaptera novaeangliae.. Hawaii Marine Mammal Commission, Washington, DC Rep.

[51] Glockner-Ferrari D, Ferrari M (1990). Reproduction in the humpback whale (Megaptera novaeangliae) in Hawaiian waters. 1975–88: the life history, reproductive rates and behaviour of known individuals identified through surface and underwater photography.. Reports of the International Whaling Commission, Special Issue.

[52] Salden DR (1988). Humpback whale encounter rates offshore of Maui, Hawaii USA.. Journal of Wildlife Management.

[53] Gabriele CM, Rickards SH, Yin SE, Frankel AS (2003). Trends in Relative Distribution, Abundance and Population Composition of Humpback Whales, Megaptera novaeangliae, in Kawaihae Bay, Hawai'i 1988–2003 Hawai'i Marine Mammal Consortium..

[54] Smultea MA (1994). Segregation by humpback whale (Megaptera novaeangliae) cows with a calf in coastal habitat near the island of Hawaii.. Canadian Journal of Zoology.

[55] Frankel AS, Clark CW (2002). ATOC and other factors affecting the distribution and abundance of humpback whales (Megaptera novaeangliae) off the north shore of Kauai.. Marine Mammal Science.

[56] Lammers MO, Au WWL, Feinholz D (2000). The Occurrence and Distribution of Marine Mammals along O ‘ahu’s Ewa/Honolulu Coast: A study to Assess Potential Interactions Between High-speed Ferry Traffic and Local Populations. MMRP.. MMRP/HIMB Technical Report.

[57] Lammers MO, Pack AA, Davis L (2007). Trends in whale/vessel collisions in Hawaiian waters.. International Whaling Commission Scientific Committee SC/59/.

[58] Szabo A, Duffus D (2008). Mother-offspring association in the humpback whale, Megaptera novaeangliae: following behaviour in an aquatic mammal.. Animal Behaviour.

[59] Cartwright R, Sullivan M (2009). Behavioral ontogeny in humpback whale (Megaptera novaeangliae) calves during their residence in Hawaiian waters.. Marine Mammal Science.

[60] Grigg RW, Grossman EE, Earle SA, Gittings SR, Lott D (2002). Drowned reefs and antecedent karst topography, Au'au Channel, S.E. Hawaiian Islands.. Coral Reefs.

[61] Erdfelder E, Faul F, Buchner A (1996). GPOWER: A general power analysis program.. Behavior research methods.

[62] Martins CCA, Morete ME, Engel MH, Freitas AC, Secchi ER (2001). Aspects of habitat use patterns of humpback whales in the Abrolhos Bank, Brazil, breeding ground.. Memoirs of the Queensland Museum.

[63] Felix F, Botero-Acosta N (2011). Distribution and behaviour of humpback whale mother and calf pairs during the breeding season off Ecuador.. Marine Ecology Progress Series.

[64] Oviedo L, Solis M (2008). Underwater topography determines critical breeding habitat for humpback whales near Osa Peninsula, Costa Rica: implications for marine protected areas.. Revista de Biologia Tropical.

[65] Sanders IM, Barrios-Santiago JC, Appeldoorn RS (2005). Distribution and relative abundance of humpback whales off western Puerto Rico during 1995–1997.. Caribbean Journal of Science.

[66] Zerbini AN, Andriolo A, Da Rocha JM, Simoes-Lopes PC, Siciliano S (2004). Winter distribution and abundance of humpback whales (Megaptera novaeangliae) off Northeastern Brazil.. Journal of Cetacean Research and Management.

[67] Blake S, Deem SL, Strindberg S, Maisels F, Momont L (2008). Roadless Wilderness Area Determines Forest Elephant Movements in the Congo Basin.. PLoS One.

[68] Eigenbrod F, Hecnar SJ, Fahrig L (2008). The relative effects of road traffic and forest cover on anuran populations.. Biological Conservation.

[69] Huijser MP, Bergers PJM (2000). The effect of roads and traffic on hedgehog (Erinaceus europaeus) populations.. Biological Conservation.

[70] McLellan BN, Shackleton DM (1988). Grizzly bears and resource-extraction industries: effects of roads on behaviour, habitat use and demography.. Journal of Applied Ecology.

[71] Constantine R (2001). Increased avoidance of swimmers by wild bottlenose dolphins (Tursiops truncatus) due to long term exposure to swim with dolphin tourism.. Marine Mammal Science.

[72] Lusseau D (2005). Residency pattern of bottlenose dolphins Tursiops spp. in Milford Sound, New Zealand, is related to boat traffic.. Marine Ecology Progress Series.

[73] Williams R, Trites AW, Bain DE (2002). Behavioural responses of killer whales (Orcinus orca) to whale-watching boats: Opportunistic observations and experimental approaches.. Journal of Zoology (London).

[74] Ciuti S, Pipia A, Ghiandai F, Grignolio S, Apollonio M (2008). The key role of lamb presence in affecting flight response in Sardinian mouflon (Ovis orientalis musimon).. Behavioural Processes.

[75] Haskell SP, Ballard WB (2008). Annual re-habituation of calving caribou to oilfields in northern Alaska: implications for expanding development.. Canadian Journal of Zoology.

[76] Lykkja ON, Solberg EJ, Herfindal I, Wright J, Rolandsen CM (2009). The effects of human activity on summer habitat use by moose.. Alces: A Journal Devoted to the Biology and Management of Moose.

[77] Elwen SH, Best PB (2004). Environmental factors influencing the distribution of southern right whales (eubalaena australis) on the south coast of South Africa I: Broad scale patterns.. Marine Mammal Science.

[78] Thomas PO (1987). Social behaviour, habitat use and interspecific interactions of southern right whale(Eubalaena australis) mother–calf pairs..

[79] Mazzuca L, Atkinson S, Nitta E (1998). Deaths and entanglements of humpback whales, Megaptera novaeangliae, in the main Hawaiian Islands, 1972–1996.. Pacific Science.

[80] Jones ME (2010). Female humpback whale (Megaptera novaeangliae) reproductive class and male-female interactions during the breeding season: Antioch University, New England.

[81] Stamation KA, Croft DB, Shaughnessy PD, Waples KA, Briggs SV (2010). Behavioral responses of humpback whales (Megaptera novaeangliae) to whale watching vessels on the southeastern coast of Australia.. Marine Mammal Science.

[82] Dunlop RA, Cato DH, Noad MJ (2008). Non song acoustic communication in migrating humpback whales (Megaptera novaeangliae).. Marine Mammal Science.

[83] Zoidis AM, Smultea MA, Frankel AS, Hopkins JL, Day A (2008). Vocalizations produced by humpback whale (Megaptera novaeangliae) calves recorded in Hawaii.. Journal of the Acoustical Society of America.

[84] Dunlop RA, Cato DH, Noad MJ (2010). Your attention please: increasing ambient noise levels elicits a change in communication behaviour in humpback whales (Megaptera novaeangliae).. Proceedings of the Royal Society B: Biological Sciences.

[85] Gibeau ML, Clevenger AP, Herrero S, Wierzchowski J (2002). Grizzly bear response to human development and activities in the Bow River Watershed, Alberta, Canada.. Biological Conservation.

[86] Singh NJ, Grachev IA, Bekenov AB, Milner-Gulland EJ (2010). Saiga antelope calving site selection is increasingly driven by human disturbance.. Biological Conservation.

[87] Craig AS, Herman LM, Pack AA (2002). Male mate choice and male-male competition coexist in the humpback whale (Megaptera novaeangliae).. Canadian Journal of Zoology.

[88] Darling JD, Jones ME, Nicklin CP (2006). Humpback whale songs: Do they organize males during the breeding season?. Behaviour.

[89] Mesnick S (1997). Sexual coercion, female mate choice and the bodyguard hypothesis: implications for the evolution of animal mating systems..

[90] Baker CS, Herman LM (1984). Aggressive behavior between humpback whales Megaptera-novaeangliae wintering in Hawaiian water USA.. Canadian Journal of Zoology.

[91] Tyack P, Whitehead H (1983). Male competition in large groups of wintering humpback whales Megaptera-noveangliae.. Behaviour.

[92] Morete ME, Bisi TL, Rosso S (2007). Temporal pattern of humpback whale (Megaptera novaeangliae) group structure around Abrolhos Archipelago breeding region, Bahia, Brazil.. Journal of the Marine Biological Association of the United Kingdom.

[93] Sundaresan SR, Fischhoff IR, Rubenstein DI (2007). Male harassment influences female movements and associations in Grevy's zebra (Equus grevyi).. Behavioral Ecology.

[94] Zerbini AN, Clapham PJ, Wade PR (2010). Assessing plausible rates of population growth in humpback whales from life-history data.. Marine Biology.

[95] Robbins J (2007). Structure and dynamics of the Gulf of Maine humpback whale population..

[96] Craig AS, Herman LM, Gabriele CM, Pack AA (2003). Migratory timing of humpback whales (Megaptera novaeangliae) in the central North Pacific varies with age, sex and reproductive status.. Behaviour.

[97] Cerchio S, Gabriele CM, Norris TF, Herman LM (1998). Movements of humpback whales between Kauai and Hawaii: Implications for population structure and abundance estimation in the Hawaiian Islands.. Marine Ecology Progress Series.

[98] Mate BR, Gisiner R, Mobley J (1998). Local and migratory movements of Hawaiian humpback whales tracked by satellite telemetry.. Canadian Journal of Zoology.

[99] Strindberg S, Buckland ST (2004). Zigzag survey designs in line transect sampling.. Journal of Agricultural Biological and Environmental Statistics.

[100] Barlow J, Forney KA (2007). Abundance and population density of cetaceans in the California Current ecosystem.. Fishery Bulletin – National Oceanic and Atmospheric Administ ration.

[101] Katona S, Baxter B, Brazier O, Kraus S, Perkins J, Winn HE, Olla BL (1979). Identification of Humpback Whales by fluke photographs.. Cetaceans, Vol.

[102] Gailey G, Ortega-Ortiz JG (2002). A note on a computer-based system for theodolite tracking of cetaceans.. Journal of Cetacean Research and Management.

[103] D.J Wright, Lundblad ER, Larkin EM, Rinehart RW (2005). http://dusk.geo.orst.edu/djl/samoa/tools.html.

[104] Tchoukanski I (2010). http://www.ian-ko.com.

[105] Beyer HL (2004). http://www.spatialecology.com/htools.

[106] McClean SA, Rumble MA, King RM, Baker WL (1998). Evaluation of resource selection methods with different definitions of availability.. Journal of Wildlife Management.

[107] Redfern JV, Ferguson MC, Becker EA, Hyrenbach KD, Good CP (2006). Techniques for cetacean–habitat modeling.. Marine Ecology Progress Series.

[108] Zar J (1999). Biostatistics, eds..

[109] Neu CW, Byers CR, Peek JM (1974). A technique for analysis of utilization-availability data.. The Journal of Wildlife Management.

[110] Hastie TJ, Tibshirani RJ (1990). Generalized additive models: Chapman & Hall/CRC.

[111] Wood SN (2006). mgcv 1.3..

[112] Zuur AF, Ieno EN, Walker NJ, Saveliev AA, Smith GM (2009). Mixed effects models and extensions in ecology with R: Springer Verlag.

[113] Burnham KP, Anderson DR (2002). Model selection and multimodel inference: a practical information-theoretic approach: Springer Verlag.

[114] Anderson DR (2008). Model based inference in the life sciences: a primer on evidence: Springer Verlag.

